# Vancomycin associated acute kidney injury in pediatric patients

**DOI:** 10.1371/journal.pone.0202439

**Published:** 2018-10-03

**Authors:** Brady S. Moffett, Jennifer Morris, Charissa Kam, Marianne Galati, Ankhi Dutta, Ayse Akcan-Arikan

**Affiliations:** 1 Texas Children's Hospital, Department of Pharmacy, Houston, Texas, United States of America; 2 Baylor College of Medicine, Department of Pediatrics, Houston, Texas, United States of America; 3 Texas Medical Center Library, Houston, Texas, United States of America; Bambino Gesù Children's Hospital, ITALY

## Abstract

**Introduction:**

Vancomycin associated acute kidney injury (vAKI) is a well known complication in pediatric patients. Identification and characterization of the incidence and risk factors for vAKI in the pediatric population would assist clinicians in potentially preventing or mitigating vAKI.

**Methods and materials:**

A 6 year retrospective cohort study was designed. Patients were included if they were < 19 years of age, received vancomycin as inpatients, and had a baseline SCr and one other SCr drawn during and up to 72 hours after the discontinuation of vancomycin. Data collection included patient demographics, vancomycin doses and length of therapy, vancomycin serum concentrations, and concomitant medications. The Kidney Disease Improving Global Outcomes (KDIGO) criteria were used to characterize acute kidney injury. Descriptive statistical methods were used and ordinal logistic regression was employed to determine variables significantly associated with vAKI.

**Results:**

A total of 7,095 patients met study criteria (55.4% male, median age 4.1 years (IQR 0.67–11.2 years)). Mechanical ventilation was used in 7.9% (n = 563) and mortality was 4.9% (n = 344). A total of 153 concomitant medications were identified. A median of 5 (IQR 3–7) SCr values were obtained and median SCr prior to vancomycin was 0.39 (IQR 0.28–0.57) mg/dL (CrCl 134±58 mL/min/1.73m^2^). Vancomycin was administered for a median of 2 (IQR 1–3) days (14.9±1.6 mg/kg/dose). vAKI was present in 12.2% (n = 862: KDIGO stage 1 (8.30%, n = 589), KDIGO stage 2 (1.94%, n = 138) KDIGO stage 3 (1.89%, n = 134)). Mean vancomycin serum concentration at 6–8 hours after a dose for patients with vAKI (10.7±8.9 mg/L) was significantly, but not clinically different for patients with no vAKI (7.5±6.3 mg/L). (p<0.05) Ordinal logistic regression identified total dose of vancomycin, vancomycin administration in the intensive care unit, and concomitant medication administration as significant for vAKI. In particular, concomitant administration of several different medications, including nafcillin, clindamycin, and acetazolamide, were noted for strong associations with vAKI. (p<0.05)

**Conclusions:**

Moderate to severe acute kidney injury due to vancomycin is infrequent in children and associated with concomitant medication use and total dose of vancomycin. Serum vancomycin concentrations are not useful predictors of vAKI in the pediatric population.

## Introduction

Vancomycin associated acute kidney injury (vAKI) is a commonly reported adverse event in pediatric patients receiving therapy with vancomycin.[1–3] Despite the widespread use of vancomycin in the pediatric population, and the known potential of nephrotoxicity, there is a lack of clarity on the etiology, true incidence, and risk factors for pediatric vAKI.[4–6] The reported incidence of vAKI ranges from 5–43% dependent on the patient population studied, the methodology used, and the criteria for vAKI.[1, 3, 5, 7–19] The effect of patient specific variables, pathophysiology, and concomitant nephrotoxic medication use can also change the reported incidence of vAKI in the pediatric population. Additionally, there are conflicting views regarding the effect of vancomycin serum concentrations on the development of vAKI in pediatric patients.[3, 12, 16, 17, 19–22]

The early identification and prevention of acute kidney injury is important as the associated morbidity and mortality are significant.[23–25] Identification of particular patient or medication variables in pediatric patients receiving vancomycin can assist clinicians in preventing vAKI and improve patient outcomes.[9, 26] An evaluation of vAKI in the pediatric population is warranted.

We plan to characterize vAKI in the inpatient pediatric population and determine the significance of patient demographic variables, vancomycin serum concentrations, and concomitant medication administration on the development of vAKI.

## Methods and materials

A retrospective, descriptive study was designed and institutional review board approval (IRB) was obtained through Baylor College of Medicine and affiliated institutions. Patients who received intravenous vancomycin at our institution were queried from the hospital electronic medical record system from January 1, 2011 –December 31, 2016. Data were fully anonymized after querying the electronic medical record system and the IRB waived informed consent. Patients were included if they received intravenous vancomycin while admitted as inpatients, were < 19 years of age, had a baseline serum creatinine (SCr) within 48 hours prior to initiation of vancomycin, and had at least one SCr drawn after initiation of vancomycin. Patients were excluded if they were undergoing renal replacement therapy at initiation of vancomycin therapy or were undergoing mechanical circulatory support. If patients had multiple admissions only the first admission was used. If multiple courses of vancomycin were prescribed, as defined by a 72 hour gap between vancomycin doses, then only data from the first course of vancomycin therapy was collected. Vancomycin is limited to 72 hours (3 days) for empiric therapy at our institution, and can be prolonged for patients with positive cultures or infectious disease consultation.

Data collection included patient demographics (age, gender, height, weight, and ethnicity), serum creatinine (SCr), vancomycin dose (mg and mg/kg), medical service, patient location in the pediatric or cardiac intensive care unit for doses of vancomycin (ICU), use of mechanical ventilation, vancomycin serum concentrations, and mortality. Concomitant medications which, based on clinical experience have been associated with acute kidney injury, included: antimicrobials, non-steroidal anti-inflammatories, vasoactive medications, diuretics, chemotherapy, calcineurin inhibitors, and radiographic contrast were recorded. Serum creatinine (SCr) values were collected as a baseline (within 48 hours prior to initiation of vancomycin) and up to 72 hours after the last dose of vancomycin therapy. Patients were considered to have undergone a cardiac surgical procedure if they underwent cardiac surgery within 7 days prior to initiation of vancomycin and cardiopulmonary bypass use was collected for these patients.

Doses of vancomycin per body weight were calculated. Body mass index and body mass index percentile (BMItile) were calculated, and obesity was defined as greater than 95^th^ percentile for age and gender.[27] Estimated creatinine clearance (CrCl) was calculated by the modified Schwartz equation.[28]

The Kidney Disease Improving Global Outcomes (KDIGO) criteria were used to determine vancomycin associated acute kidney injury (vAKI) in the patient population based on change in SCr value as compared to the lowest SCr value in the previous 72 hours.[24] (Table 1) The lowest SCr value in the prior 72 hours was used, as opposed to admission SCr, as many patients are admitted with elevated SCr due to dehydration or other illness, and this would not represent a true baseline and would potentially underestimate vAKI incidence.

**Table 1:** KDIGO criteria.

Stage	Definition
1	Increase in creatinine of ≥50% or absolute increase in creatinine of 0.3 mg/dl
2	Increase in creatinine of ≥100%
3	Increase in creatinine of ≥200% or eGFR ≥35 ml/min per 1.73 m^2^

In order to determine a true association with vAKI, vancomycin dose, concomitant medication use and vancomycin serum concentrations were collected for the 72 hours prior to maximum SCr in vAKI patients and for the entire vancomycin course for non-vAKI patients. Serum vancomycin concentrations were categorized into 6–8 hours after a dose and 11–13 hours after a dose groups and were compared between patients in each KDIGO group. For purposes of analysis, vancomycin serum concentrations < 5 mg/L (the lower limit of detection) were considered to be zero.

Descriptive statistical methods were used (mean, standard deviation, median, interquartile range (IQR), and percent) as appropriate to determine the incidence of vAKI. Student’s t-test, Wilcoxon-Rank Sum, and Fisher’s exact were used for comparison of patient demographic and clinical variables in vAKI vs non-vAKI patients. Analysis of variance (ANOVA) with post-hoc analysis and Kruskal-Wallis test were used to identify statistically significant differences between KDIGO stratifications.

Ordinal multivariable logistic regression analysis was performed to determine significant variables associated with vAKI. Included in the initial analysis were patient weight, gender, age, BMItile, days of mechanical ventilation, prior cardiac surgery and/or use of cardiopulmonary bypass, mortality, days of vancomycin therapy, dose of vancomycin (mg), number of SCr, baseline SCr, percent of doses received in the ICU, vancomycin serum concentrations at 6–8 hours after dose, and concomitant medications (antimicrobials, non-steroidal anti-inflammatories, vasoactive/inotropic medications, diuretics, chemotherapy, calcineurin inhibitors, and radiographic contrast). An automated backward stepwise analysis consisting of removal of variables with a p-value of <0.25 followed by an inclusion of values with a p-value of <0.2 was performed. All data analysis was performed with Excel 2013 (Microsoft; Redmond, Washington) and Stata IC v.12 (StataCorp; College Station, Texas). A p-value of <0.05 was considered statistically significant *a priori*.

## Results and discussion

On initial query, 23,922 patient admissions received vancomycin as inpatients at our institution. After inclusion and exclusion criteria were applied, a total of 7,095 patients met study criteria (55.4% male). The median age was 4.1 years (IQR 0.67–11.2 years) and 41.7% (n = 2949) were Hispanic. Obesity was present in 17.1% of patients 2–18 years of age. Patients were primarily admitted to pediatric hospital medicine (21.9%, n = 1550), hematology/oncology (17.9%, n = 1270), critical care medicine (12.3%, n = 872), cardiology/cardiovascular surgery (9.6%, n = 682), neonatology (8.9%, n = 633), and pulmonary medicine (4.4%, n = 311) services. Mechanical ventilation was used in 7.9% (n = 563) of patients for a median of 2 (IQR 1–3) days while patients were receiving vancomycin. Cardiovascular surgery occurred prior to vancomycin initiation in 4.8% (n = 342) of patients, and cardiopulmonary bypass was used in 1.2% (n = 122). A total of 153 concomitant medications were identified. (S1 Table). Mortality was 4.9% (n = 344).

Patients had a median of 5 (IQR 3–7) SCr values obtained during the vancomycin monitoring period (48 hours prior to vancomycin through 72 hours after discontinuation of vancomycin). Median SCr prior to vancomycin initiation was 0.39 (IQR 0.28–0.57) mg/dL and CrCl was 134±58 mL/min/1.73m^2^. Baseline SCr used for vAKI diagnosis and staging was 0.39 (IQR 0.27–0.56) and CrCl was 139±66 mL/min/1.73m^2^. Last SCr measured during the monitoring period was 0.34 (0.25–0.49) mg/dL and CrCl was 163±88 mL/min/1.73m^2^.

Patients received vancomycin for a median of 2 (IQR 1–3) days for a median of 7 (IQR 4–9) doses at a mean of 14.9±1.6 mg/kg/dose. The majority of patients (81.3%) received vancomycin for 3 days or less (as per our institutional protocol). A median 0% (IQR 0–100%) of doses of vancomycin were administered in the pediatric intensive care unit or cardiac intensive care unit, reflecting that the majority of vancomycin doses were administered outside of the intensive care unit.

A total of 4,192 serum vancomycin concentrations were drawn at a mean of 7.0±3.6 hours after a dose with a mean serum vancomycin concentration of 9.6±7.7 mg/L. When vancomycin serum concentrations were categorized, 1235 patients had one or more serum concentrations sampled between 6–8 hours after a dose (mean concentration 7.9±6.8 mg/L) and 207 patients between 11–13 hours after a dose (mean concentration 9.9±7.5 mg/L).

vAKI was present in 12.2% (n = 862) of the patient population, with KDIGO stage 1 comprising the majority of the vAKI (8.30%, n = 589), followed by KDIGO stage 2 (1.94%, n = 138) and KDIGO stage 3 (1.89%, n = 134). Comparison of patient demographic variables demonstrated statistically significant differences. (Table 2). No patients went on to renal replacement therapy due to vAKI.

**Table 2:** Comparison of patient demographic variables by AKI group.

Category (n = 7095)	No vAKI (n = 6234)	KDIGO Stage I (n = 589)	KDIGO Stage II (n = 138)	KDIGO Stage III (n = 134)
Male (%)	55.7	52.6	55.1	52.9
Age (years) Neonate (< = 30 days) (%) Infant (31 days–< 2 years) (%) Child (2–12 years) (%) Adolescent (13–18 years) (%)	6.1±5.9 10.2 31.4 39.0 19.4	6.1±5.8 1.9* 37.7* 39.2 19.4	6.8±6.2 2.2* 40.6* 31.9 25.3	6.4±6.7 18.7* 31.3 26.1* 23.9
Weight (kg)	24.5±23.5	24.0±21.5	30.0±27.9	29.3±34.5
Height (cm)	103±42	104±37	110±41	102±49
Body Mass Index (kg/m^2^) (median, IQR) Obese (%)	19.5±8.7 16.9	19.1±8.6 16.2	21.3±6.9 25.9*	22.1±9.1 22.7
Hispanic (%)	41.5	40.2	47.1	44.8
Mechanical Ventilation (%) Days of Mechanical Ventilation (median, IQR)	7.0 2 (1–2)	13.1* 1 (1–2)	16.7* 2 (1–3)	17.9* 1 (1–3)
Cardiovascular Surgical Procedure (%) Cardiopulmonary Bypass (%)	4.5 1.6	6.1 2.4	9.4* 2.9	7.5 1.5
Pre-Vancomycin SCr (mg/dL) Pre-Vancomycin CrCl (mL/min/1.73m^2^) Maximum SCr During Vancomycin (mg/dL) Minimum CrCl During Vancomycin (mL/min/1.73m^2^) Final SCr (mg/dL) Final CrCl (mL/min/1.73m^2^)	0.40 (0.29–0.57) 133±58 0.36 (0.27–0.52) 145±64 0.33 (0.25–0.48) 164±87	0.24 (0.20–0.39)* 191±83* 0.40 (0.30–0.63)* 120±52* 0.31 (0.23–0.50) 163±78	0.24 (0.15–0.46)* 201±82* 0.54 (0.35–1.02)* 91±39* 0.36 (0.22–0.72)* 167±128	0.50 (0.33–0.62) 129±128 1.05 (0.65–1.90)* 44±25* 0.86 (0.45–1.70)* 69±63*
Vancomycin Dose (mg/dose) Vancomycin Dose (mg/kg/dose) Number Vancomycin Doses (median, IQR) Vancomycin Length of Therapy (days) Percent of doses administered in ICU (median, IQR)	358±326 15.0±1.7 7 (5–10) 2 (1–3) 0 (0–100)	354±311 14.8±1.3 6 (3–9)* 2 (1–4)* 77 (0–100)	438±406* 14.6±1.4* 6 (4–9)* 2 (1–4) 86 (0–100)	394±408 14.6±1.9* 4 (3–7)* 2 (1–3) 67 (0–100)
Mortality (%)	4.2	6.3*	11.6*	23.1*

*p value < 0.05 as compared to no vAKI group

**Fig 1:**
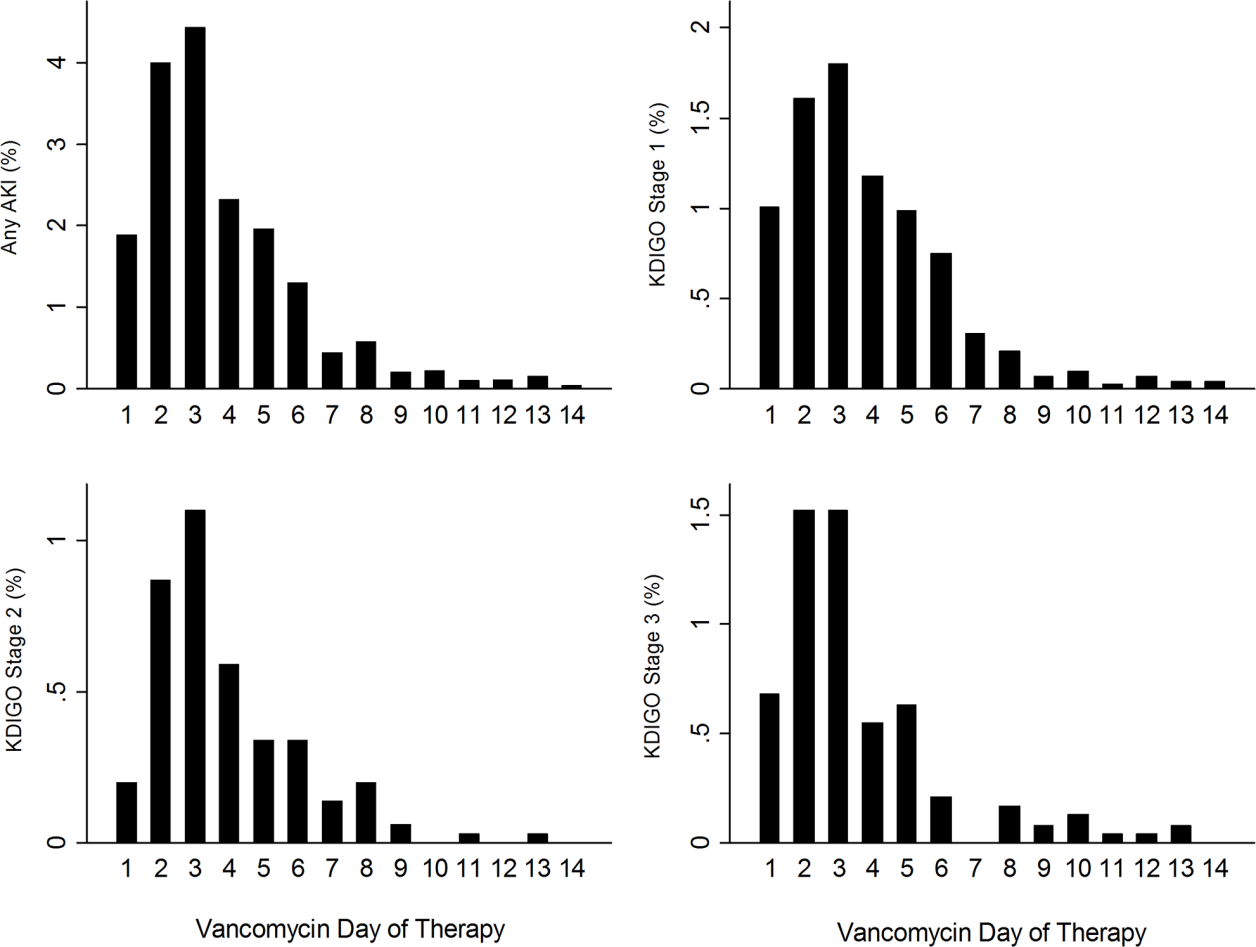
Percent of patients achieving maximum KDIGO status by vancomycin day of therapy. The incidence of AKI occurs primarily around day 3 of vancomycin therapy.

Graphs of vAKI for any KDIGO stage, and stages 1, 2, and 3 were developed to determine vAKI incidence by day of vancomycin therapy for up to 14 days of vancomycin therapy. (Fig 1) The majority of patients who experienced vAKI had maximum KDIGO stage on day 3 of vancomycin therapy. Patients who had greater than 3 days of therapy (n = 1330) had an incidence of any KDIGO stage of 19.9% (n = 264) (KDIGO Stage 1 = 14.40%, KDIGO Stage 2 = 3.01%, KDIGO Stage 3 = 2.48%).

Patients with no vAKI had a median cumulative dose of 106 (64.6–103.7) mg/kg for the entire vancomycin course of therapy, and patients with vAKI had the following cumulative dose prior to maximum KDIGO stage: Stage 1 had a median cumulative dose of 90.1 (45.1–135.5) mg/kg; Stage 2 had a median cumulative dose of 89.9 (59.4–139.3) mg/kg; Stage 3 had a median cumulative dose of 60.1 (39.9–105.2) mg/kg prior to maximum SCr value. Cumulative doses were significantly different between each KDIGO stage (p<0.05) except for between KDIGO stages 1 & 2. (p = 0.54)

All serum concentrations included were prior to maximum KDIGO status. Serum concentrations measured at 6–8 hours after a dose were significantly higher in patients with KDIGO 1, 2, and 3 as compared to patients with no vAKI (p<0.05) and serum concentrations were significantly higher in patients with KDIGO 3 as compared to patients with KDIGO 1 (p<0.05) and KDIGO 2 (p<0.05). (Table 3) In patients with serum concentrations measured at 11–13 hours after a dose, patients with KDIGO 3 had significantly higher serum concentrations than patients with no vAKI or KDIGO 1 (p<0.05). (Table 3)

**Table 3:** Serum concentrations of vancomycin at differing time periods.

KDIGO Category	6–8 hour level (n = 1235)	11–13 hour level (n = 207)
No AKI	7.8±6.5	8.7±5.9
1	9.7±8.1*	10.7±7.3
2	11.4±10.1*	11.8±6.6
3	15.3±8.9*†	19.3±10.6‡

*p<0.05 as compared to ‘No AKI’ at 6–8 hours

† p<0.05 as compared to ‘No AKI’,KDIGO category 1, or KDIGO category 2 at 6–8 hours

‡ p<0.05 as compared to ‘No AKI’ or KDIGO category 1 at 11–13 hours

Stepwise ordinal multivariable logistic regression analysis was performed and identified variables that were significantly associated with vAKI. (Table 4) The only significant patient variable for increasing odds of vAKI was admission to the intensive care unit while receiving vancomycin. (p<0.05) The dose of vancomycin (mg/dose) was significantly associated with vAKI. (p<0.05) Concomitant medications which were associated with increased odds of vAKI included: piperacillin/tazobactam, acetazolamide, sirolimus, nafcillin, alprostadil continuous infusion, colistimethate, pegaspargase, calcium chloride continuous infusion, cefoxitin, clindamycin, amikacin, and tacrolimus. (p<0.05 for all) Medications with associated with a decreased odds of vAKI included: cytarabine, gadopentetate, cefepime, and dopamine continuous infusion. (p<0.05 for all). Serum vancomycin concentrations were initially included in the model but resulted in lack of model convergence. Therefore, comparisons of patient serum vancomycin concentrations were made between patients with concomitant medications that were significant for vAKI in the multivariable model and all other patients, and these concentrations at 6–8 hours (n = 1235) (7.8±6.3 vs 7.9±7.1 mg/L, p = 0.79) and 11–13 hours (n = 207) (10.5±8.5 vs 9.2±6.9 mg/L, p = 0.26) were not significantly different between these two patient groups.

**Table 4:** Ordinal logistic regression model for vancomycin associated acute kidney injury.

Variable (n = 7095) (% of patients)	Odds Ratio	p value	95% CI
Dose of Vancomycin (mg)	1.001	<0.001	1.0008–1.001
Cytarabine (2.8%)	0.325	<0.001	0.177–0.595
Piperacillin/Tazobactam (25.3%)	1.458	0.001	1.156–1.836
Acetazolamide (1.6%)	3.192	0.003	1.489–6.838
Sirolimus (0.31%)	5.199	0.006	1.619–16.69
Nafcillin (2.4%)	2.209	0.008	1.226–3.978
Alprostadil (continuous infusion) (0.85%)	3.555	0.011	1.343–9.407
Gadopentetate (6.4%)	0.543	0.011	0.338–0.869
Cefepime (5.9%)	0.531	0.012	0.325–0.868
Colistimethate (0.24%)	4.872	0.013	1.404–16.90
Pegaspargase (0.51%)	3.239	0.015	1.258–8.340
Calcium Chloride (continuous infusion) (0.52%)	5.043	0.020	1.286–19.77
Cefoxitin (0.17%)	4.753	0.024	1.224–18.45
Dopamine (continuous infusion) (7.03%)	0.563	0.026	0.339–0.932
Doses Received in the ICU (48.4%)	1.003	0.028	1.000–1.005
Clindamycin (6.9%)	1.510	0.033	1.034–2.204
Amikacin (0.27%)	4.174	0.046	1.023–17.02
Tacrolimus (3.34%)	1.777	0.046	1.010–3.126
Ganciclovir (1.3%)	2.256	0.051	0.997–5.102
Lisinopril (0.34%)	3.210	0.054	0.979–10.52
Iohexol (5.2%)	0.669	0.059	0.441–1.015
Vasopressin (continuous infusion) (2.0%)	0.493	0.068	0.231–1.053
Albumin (8.3%)	1.368	0.069	0.976–1.917
Labetalol (0.24%)	2.983	0.070	0.913–9.738
Penicillin G (0.59%)	2.289	0.081	0.901–5.810
Chlorothiazide (3.4%)	1.740	0.098	0.902–3.355
Micafungin (0.58%)	0.306	0.104	0.073–1.276
Dapsone (0.11%)	4.482	0.105	0.731–27.46
Furosemide (22.7%)	0.800	0.106	0.610–1.048
Ibuprofen (9.51%)	1.315	0.111	0.939–1.841
Acyclovir (7.9%)	1.373	0.114	0.926–2.036
Fludarabine (0.45%)	0.298	0.115	0.066–1.342
Daunorubicin (1.5%)	0.482	0.127	0.188–1.231
Atovaquone (0.16%)	4.502	0.128	0.649–31.21
Losartan (0.13%)	3.545	0.137	0.667–18.83
Doxorubicin (0.18%)	0.162	0.141	0.014–1.824
Methotrexate (0.51%)	0.415	0.145	0.127–1.354
Epinephrine (continuous infusion) (3.8%)	1.423	0.156	0.874–2.316
Vincristine (2.5%)	0.595	0.159	0.288–1.225
Nitroprusside (continuous infusion) (1.38%)	0.546	0.165	0.232–1.282
Fluconazole (4.8%)	1.334	0.167	0.886–2.004
Linezolid (0.13%)	4.726	0.170	0.514–43.44
Cidofovir (0.03%)	0.106	0.178	0.004–2.785
Valganciclovir (0.82%)	0.501	0.204	0.172–1.456
Amphotericin B (non-lipid) (0.96%)	1.563	0.223	0.761–3.206
Caspofungin (0.69%)	0.505	0.223	0.168–1.513
Ciprofloxacin (1.62%)	1.473	0.239	0.772–2.809
Esmolol (0.83%)	0.463	0.241	0.127–1.677

We have reported the largest cohort of pediatric patients receiving vancomcyin to date examining the incidence and factors associated with vAKI. The incidence of vAKI was 12.2% in our population, and for those patients receiving more than 3 days of vancomycin was 19.9%, which is similar to other reports of vAKI in the pediatric population.[3, 29] It should be noted that moderate vAKI (KDIGO Stage 2) and severe vAKI (KDIGO Stage 3) were infrequent, with only 1.84% and 1.89% of the study population, respectively, with no patients proceeding on to renal replacement therapy after vancomycin therapy was completed. Based upon these data, we would suggest that the incidence of moderate to severe vAKI is low in the pediatric population and occurs early at approximately 72 hours of vancomycin therapy.

The multivariable analysis identified variables that should be considered for future investigations and current patient care. Patient variables, including body habitus, patient weight, and age, were not significant for vAKI though prior publications have associated obesity with increased incidence of vAKI.[30] Our previous investigations have identified that obese patients do not have higher serum concentrations of vancomycin, but would have a greater total exposure of vancomycin when dosed by body weight.[31] Dose of vancomycin was significant, which suggests that greater individual doses of vancomycin may be a more significant factor for vAKI as compared to serum concentrations, which has been noted in prior publications.[18] This suggests that higher individual doses of vancomycin are more likely to lead to vAKI, independent of serum concentrations.

The serum vancomycin concentrations identified that were associated with KDIGO Stages were much lower than what is typically considered as a risk factor for vAKI. Although the differences were significant, the mean vancomycin serum concentration value at 6–8 hours after a dose (which would be a typical time for monitoring of trough concentrations) for a patient with KDIGO Stage 1 was 9.7±8.1 mg/L and for KDIGO Stage 2 was 11.4±10.1 mg/L. Both of these mean values have a wide standard deviation and are considerably lower than the often cited 15 mg/L for prevention of vAKI.[12, 21, 32–34] We sought to minimize the concerns of causation versus outcome by collecting serum vancomycin concentrations in the 72 hours prior to vAKI, and to collect values at particular times after a dose, rather than a trough concentration, to capture data from those patients who may have been receiving non-scheduled vancomycin dosing due to real or perceived pre-existing kidney dysfunction.[20] Therefore, we were unable to evaluate serum concentrations at specifically at steady state, and values sampled at steady state may be different than what we have been able to capture.

Strategies to prevent vAKI should likely not focus on serum concentration monitoring but on other related factors. A vancomycin serum concentration of ~10 mg/L has been cited as the goal concentration to achieve an AUC:MIC ratio of >400 in the pediatric population for efficacy.[35] This serum concentration is similar to the mean concentrations identified in our analysis at 6–8 hours and 11–13 hours after a dose, in patients with and without vAKI. Reduction in serum concentrations to prevent vAKI would risk therapeutic failure and undesirable clinical outcomes. Vancomycin serum concentrations do not appear to be directly related to vAKI and 15 mg/L as a breakpoint for vAKI does not appear to be warranted. We would advocate monitoring of SCr or other novel kidney injury biomarkers as opposed to serum vancomycin concentration monitoring to prevent or minimize vAKI in pediatric patients receiving vancomycin.

Several expected variables were associated with vAKI, as reported by other investigators, and give credence to the analysis. Receipt of vancomycin in the intensive care unit was not unexpectedly related to vAKI, as was the concomitant use of vasoactive medications such as calcium chloride continuous infusion and alprostadil continuous infusion. The impact of intensive care unit admission was likely mitigated by the incorporation of variables typically associated with critical illness, such as mechanical ventilation, cardiac surgery, and vasoactive medication use. These medications would primarily be used in patients with hemodynamic instability or low cardiac output, particularly the neonatal population, and could be considered surrogate markers for decreased kidney perfusion. The use of known nephrotoxic medications, such as sirolimus, colistimethate, tacrolimus, and amikacin were identified in our analysis and would be expected to increase the incidence of vAKI in patients receiving these medications concomitantly with vancomycin.[36] Other medications noted as significant that would not typically be known to contribute to vAKI were cefoxitin and pegaspargase, but these were present in very small numbers with wide confidence intervals, and their impact on vAKI is questionable and should be interpreted with caution.

Unique findings to our analysis include the association of other antimicrobials with vAKI. The multivariable analysis identified the significance of concomitant use of piperacillin/tazobactam (p = 0.001), nafcillin (p = 0.008), and clindamycin (p = 0.033) use of with vAKI. The association of other antimicrobials with vAKI that are not typically considered nephrotoxic should not be immediately discounted or speculated to have been due to underlying patient factors and pathophysiologies that were not captured in the multivariable analysis and require further study.[37] The analysis we have performed is in agreement with recent reports of piperacilln/tazobactam associations vAKI, which has only been recently identified.[38, 39] The strength of the associations of nafcillin and clindamycin with vAKI warrant that the use of nafcillin or clindamycin with vancomycin should be investigated further.

Conspicuously non-significant is concomitant aminoglycoside use (excepting the small number of patients who received amikacin) as associated with vAKI. Prior publications have noted that in neonates the combination of gentamicin and vancomycin was no more nephrotoxic than either agent as monotherapy, while data in adults has demonstrated that combination therapy was more likely to lead to acute kidney injury.[40, 41] Practitioners may have been more vigilant in patients who were receiving both medications and limited exposure to both to prevent acute kidney injury. Similarly, the protective effect of cefepime and cytarabine is likely related to institutional protocols and provider practices. Cefepime and vancomycin are first line agents for febrile neutropenia at our institution, and are administered with aggressive hydration, which could potentially decrease SCr concentrations.

The concomitant use of dopamine was also idenitified to have a protective effect for vAKI. The majority of published data, which is primarily in adults, has concluded that dopamine does not prevent acute kidney injury or hasten the recovery period after acute kidney injury.[42, 43] However, other publications have described an increase in urine output in patients receiving dopamine.[44] We have previously described the ability of fenoldopam, a dopamine receptor agonist, to increase urine output in critically ill patients.[45] There is theoretical potential for increased clearance of vancomycin, and decreased risk of vAKI, in patients who have received dopamine, but this is only speculation and would need to be validated.

Unusual to our analysis is the finding that concomitant acetazolamide use had a strong association with vAKI. Acetazolamide inhibits carbonic anhydrase in the renal tubule, reverses secretion of hydrogen ions, alkalinizes urine, and increases the secretion of sodium, potassium, bicarbonate, and water.[46] The mechanism for vAKI is not completely understood, though current literature supports the concept of free radical generation, oxidative phosphorylation, mitochondrial dysfunction, and cellular apoptosis in the lumen of the kidney.[6] The etiology of vAKI in the presence of acetazolamide should be investigated.

The limitations to this review are those germane to all retrospective evaluations and thorough evaluation of these results prior to application in practice is necessary. We believe that our analysis is representative of the inpatient pediatric population, but do realize that this is a single center evaluation and practices in dosing, monitoring, and empiric use of vancomycin will vary depending on institution. We did not evaluate intensity of nephrotoxic medication exposure, which has been shown to increase the incidence of acute kidney injury in pediatric patients, as one of the goals of the manuscript was to identify individual medications which are associated with vAKI.[47] While SCr monitoring is a common clinical practice at our institution in patients receiving vancomycin, we have likely selected for higher acuity patients who had more frequent SCr monitoring. The types of medications and dosing strategies for these medications used concomitantly with vancomycin will also vary at differing institutions with different formularies. We also recognize that we only used SCr as a marker of vAKI and addition of urine output would likely capture a higher incidence of vAKI.[25]

We did not include a control group of patients who were not exposed to vancomycin for comparison due to the potential for confounding by indication. Patients who receive vancomycin would potentially be at higher risk of developing acute kidney injury, than those who did not receive vancomycin, based on underlying pathophysiologies and indication for vancomycin. Evaluation of patient pathophysiology is critical in determining the risk of developing vAKI and should be part of the strategy for pharmacotherapy with vancomycin. Further investigations into vAKI in the pediatric patient population should focus on underlying pathophysiologies and characterize vAKI using newer technologies and biomarkers.

## Conclusions

Moderate to severe acute kidney injury associated with vancomycin is infrequent in the pediatric population and is often associated with critical illness, concomitant medication administration, and total amount of vancomycin exposure. Monitoring of serum vancomycin concentrations to prevent acute kidney injury is of questionable benefit.

## Supporting information

S1 Table Concomitant medications. Medications prescribed during vancomycin therapy.
(DOCX)
