# Supplementary material for: Vancomycin associated acute kidney injury in pediatric patients
Source: PLoS One. 2018 Oct 3;13(10):e0202439. doi: 10.1371/journal.pone.0202439 (PMC6169857; doi:10.1371/journal.pone.0202439)
Supplement: S1 Table — Medications prescribed during vancomycin therapy. (DOCX) [file pone.0202439.s001.docx]

**Appendix I**

**Concomitant Medications (n=7095) (%)**

| Acetazolamide (1.6) | Acyclovir (7.95) | Albumin (8.25) | Alprostadil (0.85) | Ambrisentan (0.03) | Atovaquone (0.16) |
| --- | --- | --- | --- | --- | --- |
| Sulfamethoxazole/  Trimethoprim (7.4) | Bosentan (0.20) | Bumetanide (1.00) | Captopril (0.63) | Carvedilol (0.42) | Losartan (0.13) |
| Chlorothiazide (3.4) | Cidofovir (0.03) | Ciprofloxacin (1.62) | Clindamycin (6.88) | Clonidine (1.21) | Sildenafil (2.02) |
| Furosemide (22.7) | Ganciclovir (1.27) | Gentamicin (7.77) | Hydralazine (0.76) | Hydrochlorothiazide (0.18) | Phenylephrine (continuous infusion) (0.06) |
| Metoprolol (0.47) | Metronidazole (6.24) | Micafungin (0.58) | Tobramycin (1.21) | Nadolol (0.06) | Cyclophosphamide (1.61) |
| Stavudine (0.01) | Ketorolac (4.1) | Ticarcillin/  Clavulanate (0.10) | Nitrofurantoin (0.13) | Valacyclovir (0.21) | Melphalan (0.01) |
| Naproxen (0.18) | Dexrazoxane (0.01) | Nitazoxanide (0.03) | Doxorubicin (0.18) | Nitroglycerin (continuous infusion) (0.08) | Iohexol (5.21) |
| Daunorubicin (1.45) | Pegasparaginase (0.51) | Sorafenib (0.01) | Thiotepa (0.01) | Erwinia (0.03) | Azithromycin (7.24) |
| Mitoxantrone (0.03) | Amphotericin B (non-lipid) (0.96) | Ampicillin (1.76) | Ampicillin/  Sulbactam (0.08) | Anidulafungin (0.06) | Ceftriaxone (10.27) |
| Amoxicillin/  Clavulnate (0.17) | Cefotaxime (27.53) | Cefoxitin (0.17) | Cefpodoxime (0.06) | Esmolol (continuous infusion) (0.83) | Fluconazole (4.79) |
| Enalapril (2.23) | Enalaprilat (0.17) | Epoprostenol (0.21) | Erythromycin (0.58) | Lisinopril (0.34) | Meropenem (0.85) |
| Ketoconazole (0.01) | Labetalol (0.24) | Levofloxacin (0.39) | Linezolid (0.13) | Propranolol (0.87) | Sotalol (0.24) |
| Oseltamivir (5.3) | Penicillin G (0.59) | Piperacillin/  Tazobactam (25.3) | Posaconazole (0.13) | Milrinone  (continuous infusion) (4.55) | Vasopressin  (continuous infusion) (2.00) |
| Calcium Chloride (continuous infusion) (0.52) | Dobutamine (continuous infusion) (0.13) | Dopamine (continuous infusion) (7.03) | Epinephrine (continuous infusion) (3.83) | Cisplatin (0.10) | Cytarabine (2.83) |
| Bleomycin (0.06) | Gadofosveset (0.04) | Busulfan (0.03) | Carboplatin (0.01) | Leucovorin (0.27) | Mesna (1.65) |
| Imatinib (0.06) | Amlodipine (2.16) | Gadopentetate (6.36) | Lestaurtinib (0.01) | Gadoteridol (0.01) | Iothalamate (0.80) |
| Fludarabine (0.45) | Cefepime (5.95) | Amikacin (0.27) | Gadoterate (1.45) | Atenolol (0.59) | Clofarabine (0.01) |
| Amiloride (0.01) | Dexmedetomidine (6.46) | Caspofungin (0.69) | Aztreonam (0.03) | Ceftazidime (10.08) | Leuprolide (0.08) |
| Cefazolin (1.59) | Ivermectin (0.01) | Colistimethate (0.24) | Cefuroxime (0.44) | Ethacrynic Acid (0.20) | Gadoxetate (0.06) |
| Dapsone (0.11) | Nifedipine (0.72) | Imipenem (0.08) | Foscarnet (0.08) | Lopinavir/Ritonavir (0.03) | Rifampin (0.63) |
| Isoproterenol (0.16) | Vincristine (2.51) | Nafcillin (2.41) | Metolazone (0.54) | Norepinephrine (continuous infusion) (1.79) | Dasatinib (0.10) |
| Nicardipine (continuous infusion) (0.04) | Asparaginase (0.01) | Valganciclovir (0.82) | Spironolactone (2.41) | Nitroprusside (continuous infusion) (1.38) | Methotrexate (0.51) |
| Voriconazole (1.30) | Vinorelbine (0.01) | Ifosfamide (0.10) | Ibuprofen (9.510 | Etoposide (0.63) | Ioversol (6.31) |
| Idarubicin (0.04) | Cyclosporine (1.30) | Tacrolimus (3.34) | Sirolimus (0.31) |  |  |
